# Performance of an AI-powered visualization software platform for precision surgery in breast cancer patients

**DOI:** 10.1038/s41523-024-00696-6

**Published:** 2024-11-14

**Authors:** Michelle Weitz, J. R. Pfeiffer, Snehal Patel, Matthew Biancalana, Arda Pekis, Vignesh Kannan, Evandros Kaklamanos, Amanda Parker, Jesse E. Bucksot, José Rubio Romera, Ryan Alvin, Yuhan Zhang, Andrew T. Stefka, Dorys Lopez-Ramos, Joseph R. Peterson, Anuja K. Antony, Kathryn W. Zamora, Stefanie Woodard

**Affiliations:** 1 SimBioSys, Inc., Chicago, IL USA; 2 Department of Radiology, University of Alabama at Birmingham School of Medicine, Birmingham, AL USA

**Keywords:** Surgical oncology, Breast cancer

## Abstract

Surgery remains the primary treatment modality in the management of early-stage invasive breast cancer. Artificial intelligence (AI)-powered visualization platforms offer the compelling potential to aid surgeons in evaluating the tumor’s location and morphology within the breast and accordingly optimize their surgical approach. We sought to validate an AI platform that employs dynamic contrast-enhanced magnetic resonance imaging (DCE-MRI) to render three-dimensional (3D) representations of the tumor and 5 additional chest tissues, offering clear visualizations as well as functionalities for quantifying tumor morphology, tumor-to-landmark structure distances, excision volumes, and approximate surgical margins. This retrospective study assessed the visualization platform’s performance on 100 cases with ground-truth labels vetted by 2 breast-specialized radiologists. We assessed features including automatic AI-generated clinical metrics (e.g., tumor dimensions) as well as visualization tools including convex hulls at desired margins around the tumor to help visualize lumpectomy volume. The statistical performance of the platform’s automated features was robust and within the range of inter-radiologist variability. These detailed 3D tumor and surrounding multi-tissue depictions offer both qualitative and quantitative comprehension of cancer topology and may aid in formulating an optimal surgical approach for breast cancer treatment. We further establish the framework for broader data integration into the platform to enhance precision cancer care.

## Introduction

Breast cancer is the most common solid-tumor cancer worldwide, with 1 in 8 U.S. women developing invasive breast cancer over the course of their lifetime^1^. Surgery persists as the main treatment modality in the management of early-stage invasive breast cancer^2^. Breast-conserving surgery (BCS) is an area of growing interest for the treatment of breast cancer, where it has been shown to have physical and psychological benefits that improve patient satisfaction and quality of life^3^. The surgical landscape has been transformed by numerous clinical trials that have demonstrated that BCS can offer reduced morbidity and improved cosmetic outcomes for women, without compromising long-term survival^4–7^ or satisfaction^8^.

Many factors unique to individual patients^9^, such as medical comorbidities, play a critical role in influencing treatment modality. However, there remain key spatial aspects of the tumor influencing operative decisions, including tumor size, proximity to local chest structures, multifocality, excision volume, tumor-to-breast-volume ratio, and gland density^2^. The relationship of the tumor to the nipple and skin are of particular importance when considering nipple- and skin-sparing mastectomy, respectively, and the oncological safety of immediate breast reconstruction^10^. Moreover, the relationship of the tumor to the chest wall is relevant when weighing breast conservation and mastectomy^11^. Even the width of surgical margins represents an important decision point for surgeons. Margins that are too wide may yield poor cosmetic results^12^, while margins that are too narrow risk incomplete resection and disease recurrence^13^.

Despite significant progress, re-excision rates secondary to positive margins nevertheless remain at levels roughly double^14,15^ the 10% goal set by the American Society of Breast Surgeons^16,17^. When formulating a surgical plan, the surgeon is challenged with integrating key features of the patient’s anatomy, as well as tumor and tumor microenvironment features in multiple dimensions (spatially, temporally, metabolically, etc.), to mentally approximate the tumor location, size, and the overall extent of disease. One way for surgeons to visualize these features is dynamic contrast-enhanced magnetic resonance imaging (DCE-MRI), which over the last decade has emerged as a standard for breast imaging to aid in the assessment of these morphometric dimensions^18^. Not only does DCE-MRI yield more precise tumor characterization compared to mammography, but this modality also allows for the visualization of tumor morphology in the context of breast, chest wall, and adjacent structures in three-dimensional (3D) space^18^.

Although DCE-MRI alone offers enhanced capabilities over standard MRI practices, further improvement in visualization and in assessing tumor characteristics are achievable by computational spatial analysis of the DCE-MRI data. The medical imaging field broadly refers to the identification and mapping of these specific tissue types as “segmentation” – delimiting the boundaries between distinct anatomical features, such as tumor, blood vessels, or bone. These models can facilitate the planning of the surgical approach, teaching of surgery residents, and communicating with patients. These models have proven especially valuable for planning surgeries in which the preservation of normal parenchyma is critically important for maintaining quality of life and physiologic function in a variety of tumor types^19–22^. 3D models can also help map the tumor compartment to its vascular supply, thereby aiding in the surgical planning of resection margins that are dictated by the organ’s regional arterial territory^23,24^. Importantly, studies that examined clinical outcomes resulting from surgeries performed using 3D modeling guidance report early evidence of improved surgical results and patient benefit^25^, including in randomized clinical trials^26^.

Here, we investigate the performance and utility of TumorSight Viz and its corresponding data analysis pipeline, which together provide detailed 3D spatial insights into anatomical structures, landmarks, and their relative positioning based on data extracted from routine standard-of-care DCE-MRI^27^ (Supplementary Fig. 1). TumorSight Viz is an FDA-cleared medical device that assists in clinical decision-making for early-stage, biopsy-proven breast cancer, prior to neoadjuvant therapy. The platform generates 3D visualization maps of breast tumors alongside 5 additional tissue types, including fibroglandular breast tissue, adipose tissue, skin, chest wall, and blood vessels^27^. TumorSight Viz provides a surgical roadmap of key anatomical landmarks as well as qualitative visualizations, and further defines automated quantitative assessments such as tumor-to-landmark distances and breast quadrant localization. We assessed the platform’s performance against board-certified radiologists on a single institution cohort (*n* = 100). We show that TumorSight Viz segmentations and related surgical margin visualizations are comparable to those approved by experts, and that its automated methods for tumor and tumor-to-landmark measurements are accurate.

## Results

### Datasets for assessment of TumorSight Viz performance

**Fig. 1:**
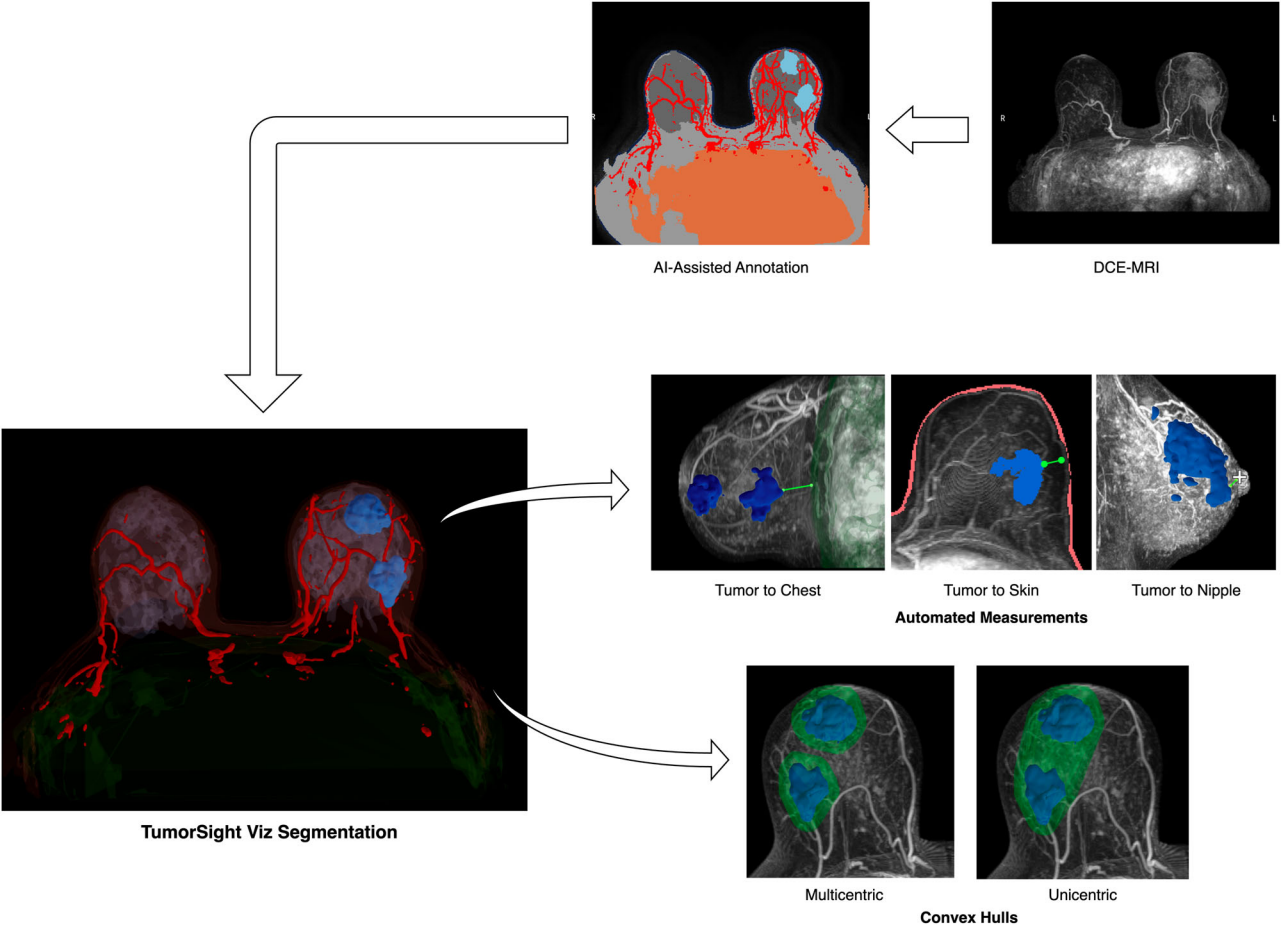
TumorSight Viz workflow. (**left**) TumorSight Viz *visualization* shows the 3D rendering of multifocal breast cancer (blue) along with breast fibroglandular (lavender) and adipose (brown) tissue, skin (peach), blood vessels (red), chest (green) and nipple (+). (**right-top**) 2D view of raw MRI and TumorSight Viz segmentation demonstrates accurate segmentation of breast tumors and other tissues. (**right-mid**) The *clinical metrics* functionality uses chest (green), skin (peach), and nipple (+) labels to measure closest approach of the tumor to relevant tissues. (**right-bottom**) The *convex hull* feature accurately delineates hypothetical surgical margins (green) for piecemeal (multicentric) and *en bloc* (unicentric) resections of multifocal tumors.

**Fig. 2:**
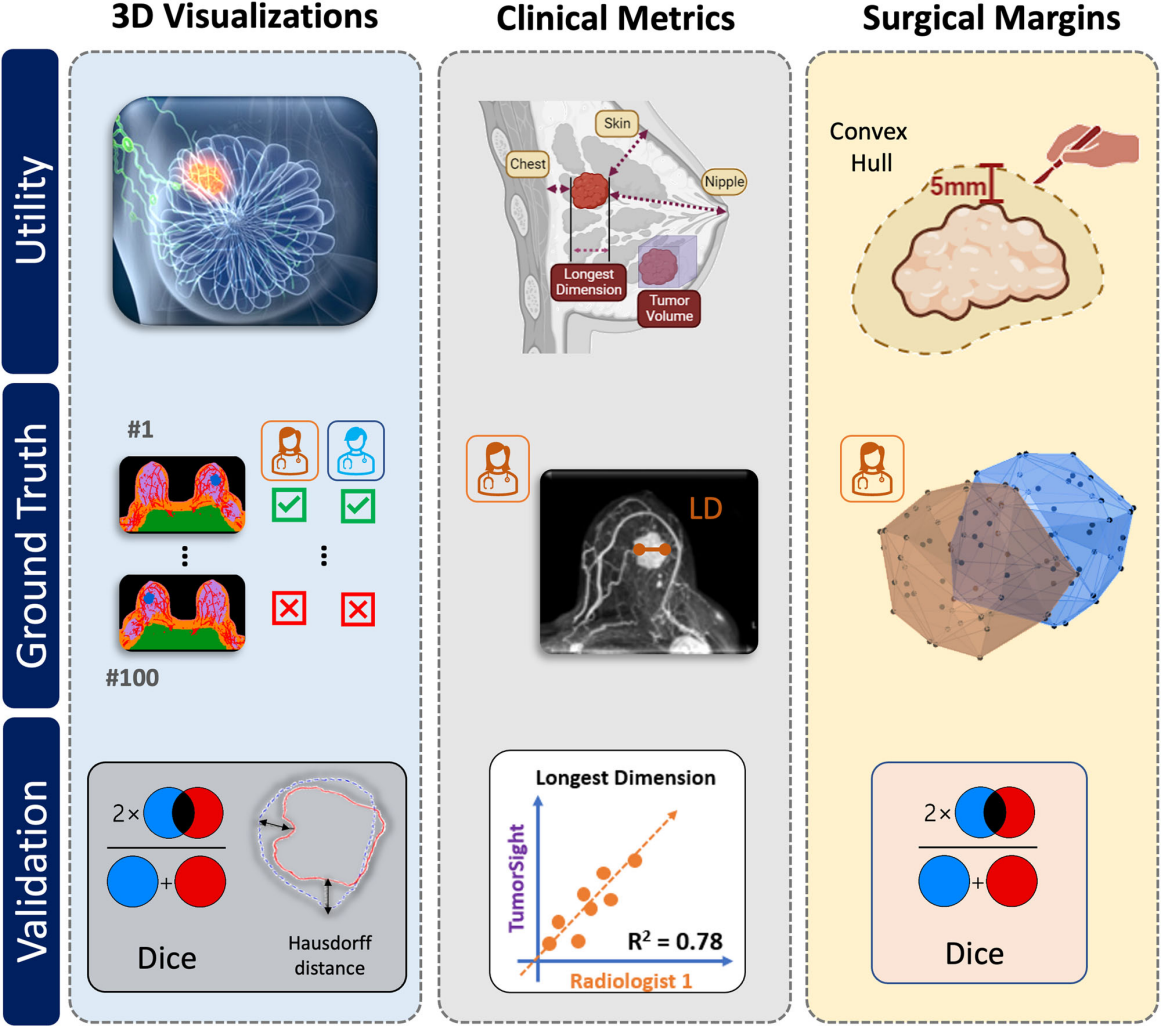
Study roadmap. TumorSight Viz clinical utility **(top)** was extended beyond 3D tumor and tissue visualizations. Functionality added in this study includes automated measurements of tumor dimensions, tumor-to-landmark distances, and breast quadrant localization and automated generation of a convex hull set at a desired distance from the tumor to approximate surgical margins. The ground-truth (**middle**) for validating TumorSight Viz 3D visualizations was composed of annotations approved by 2 radiologists. The automated clinical metrics were validated against either direct manual measurements made by 2 radiologists or metrics automatically extracted from the radiologist-approved annotations. Radiologist-approved tumor annotations were also used to generate ground-truth convex hulls. A suitable evaluation metric (**bottom**) was selected for the validation of each TumorSight Viz functionality. This figure contains images derived under the Creative Commons License.

To assess TumorSight Viz performance, we assembled a cohort of breast cancer patients whose data was not used during the training and testing phases of TumorSight Viz development. This cohort therefore represents a truly independent validation set. The subjects in this cohort were selected via a retrospective search of the hospital radiology database for women with early-stage breast cancer who had diagnostic DCE-MRIs between January 1, 2001 and December 31, 2020. Here, “early stage” was defined as having no clinical or radiologic evidence of distant metastatic disease at the time of treatment initiation (i.e., the patients were cM0 at the time of treatment initiation). Patients who completed standard-of-care neoadjuvant chemotherapy and subsequently underwent surgery for pathologic evaluation of treatment response were included. Patients whose MRIs had major artifacts or other imaging issues were excluded. Examples of issues that warranted exclusion are lack of fat suppression, incomplete DICOM series, and non-correctable registration errors. The age at diagnosis, race, ethnicity, cancer subtype, clinical T stage, and clinical N stage were abstracted from the hospital medical records, when available. Our work complies with the Checklist for Artificial Intelligence in Medical Imaging guidelines prescribed by the Radiological Society of North America^28^. An overview of our study is provided in Figs. 1 and 2. An overview of the core segmentation model development (described extensively elsewhere^27^) is provided in Supplementary Fig. 1, and statistical analysis of these datasets is provided in Table 1.

**Table 1:** Cohort demographic and clinical characteristics

	Train (*N* = 358)	Test (*N* = 46)	UAB (*N* = 100)
Age			
Mean (SD)	50.4 (11.5)	53.5 (12.3)	50.1 (12.2)
Median [Min, Max]	50.0 (23.0, 85.0)	55.1 (23.0, 77.0)	48.3 (20.3, 83.6)
Missing	2 (0.6%)	0 (0%)	0 (0%)
Race/Ethnicity			
African American	66 (18.4%)	8 (17.4%)	25 (25.0%)
Asian	17 (4.7%)	4 (8.7%)	3 (3.0%)
Caucasian	246 (68.7%)	32 (69.6%)	70 (70.0%)
Hispanic	1 (0.3%)	0 (0%)	0 (0%)
Native American	5 (1.4%)	0 (0%)	0 (0%)
Other	8 (2.2%)	0 (0%)	0 (0%)
Missing	15 (4.2%)	2 (4.3%)	2 (2.0%)
Cancer Subtype			
IDC	187 (52.2%)	22 (47.8%)	80 (80.0%)
ILC	8 (2.2%)	1 (2.2%)	12 (12.0%)
Other	160 (44.7%)	23 (50.0%)	8 (8.0%)
T Stage			
T1	56 (15.6%)	3 (6.5%)	17 (17.0%)
T2	187 (52.2%)	33 (71.7%)	54 (54.0%)
T3	105 (29.3%)	9 (19.6%)	28 (28.0%)
T4	9 (2.5%)	1 (2.2%)	1 (1.0%)
Missing	1 (0.3%)	0 (0%)	0 (0.0%)
N Stage			
N0	102 (28.5%)	10 (21.7%)	19 (19.0%)
N1	84 (23.5%)	12 (26.1%)	15 (15.0%)
N2	15 (4.2%)	2 (4.3%)	5 (5.0%)
N3	3 (0.8%)	0 (0%)	0 (0%)
NX	1 (0.3%)	0 (0%)	1 (1.0%)
Missing	153 (42.7%)	22 (47.8%)	60 (60.0%)

*IDC* Invasive Ductal Carcinoma, *ILC* Invasive Lobular Carcinoma, *Other* Ductal Carcinoma in situ, Invasive Mammary Carcinoma.

### Patient characteristics in the validation study

Our validation set was composed of 98 women comprising 100 cases (in which 2 women presented with bilateral breast cancer, see *Methods – Sample Size Determination and Acceptance Criteria*) (Table 1). The validation set was a random subsample of a patient cohort from the University of Alabama (UAB), obtained under a waiver from the organization’s Institutional Review Board (IRB). The sampling was balanced with respect to estrogen receptor status, progesterone receptor status, human epidermal growth factor receptor status, grade, T stage, laterality, tumor volume, tumor longest dimension, and MRI resolution within the overall UAB cohort (not the overall dataset). Each woman in this set had a confirmatory bilateral DCE-MRI and completed a neoadjuvant chemotherapy regimen at UAB. In each validation set, the average age at diagnosis was 50 years. The distribution by race was 70% white, 25% black, and 3% Asian.

Invasive ductal carcinoma (IDC) was by far the predominant histologic type, accounting for 80% of tumors in the validation cohort. Most women presented at clinical tumor stage of cT3 and clinical nodal stage of cN0. Tumor and multi-tissue labels were generated in TumorSight Viz using an AI labeling procedure for all DCE-MRIs. The performance of these automatic labels and metrics was then assessed relative to metrics determined by practicing radiologists.

### Summary of methodology

Our validation methodology necessitated complete, voxel-level labeling of DCE-MRIs encompassing 6 different tissues and air. Because making these assignments would have been a Herculean task for two practicing radiologists to perform on a large patient reference dataset, we instead used physician experts to approve labels produced by trained lay people, as performed in previous validation studies^29^. Additionally, tumor dimensions automatically extracted from radiologist-approved segmentations correlated well with direct measurements made by the radiologists. This similarity was reflected in comparable correlations between radiologist vs. TumorSight Viz segmentations as compared to inter-radiologist correlations. Collectively, these findings verify the high quality of radiologist-approved annotations and support their use as proxies for radiologist-generated annotations.

### Automated nipple localization

The nipple is a critical surgical landmark, serving as a key point of reference in terms of breast surgery type and feasibility assessment^30^. Using TumorSight Viz 3D renderings, we developed a simple heuristic for localizing a single voxel representing the nipple. For each breast, we first find the center of mass of all voxels laying within 8 mm of at least one voxel each of air, skin, and fibroglandular tissue, and then offset this point by 6 mm in the posterior direction.

### Clinical metrics calculations

Having established the ability to label key landmarks, including skin, chest, and nipple, we introduced additional algorithms into TumorSight Viz for automating clinically-relevant measurements that can provide surgeons with a quantitative understanding of tumor topology and tumor-landmark distances^27^.

### Axis-aligned tumor dimensions

We automated calculation of the longest axis-aligned tumor dimension and tumor bounding box volume by first computing the longest tumor dimension in the transverse, coronal, and sagittal planes using the tumor label map. The longest axis-aligned dimension is the maximum of those dimensions, and the bounding box volume is their product. The longest axis-aligned dimension and bounding box volume was calculated for the entire tumor, as well as for the dominant tumor mass. The dominant mass was operationally defined as the largest contiguous collection of “tumor” voxels. These measurements are expected to be identical for unifocal tumors but different for multifocal tumors.

To assess relative tumor volume (e.g., as a percent of total breast volume), we implemented a series of volume calculation algorithms. The tumor volume algorithm first counts the tumor voxels and then multiplies by the voxel volume. The breast volume algorithm first computes a “breast box” (see *Methods, Breast Box Construction*) around the breast and then sums voxel volumes of all valid breast tissues (i.e., skin, adipose tissue, fibroglandular tissue, blood vessels, and tumor) within the breast box.

### Tumor-to-landmark distances

The automatic tumor-to-landmark distance calculations were made by first extracting the coordinates of the vertices of each voxel of the tumor and landmark. The shortest distance between the tumor and landmark vertices, as well as the tumor-to-skin, tumor-to-nipple, and tumor-to-chest distances, were computed using the KDTree class, as implemented in the SciPy Python package.

### Breast quadrant localization

The breast quadrant localization algorithm partitions the breast into 4 quadrants with equal volumes of breast tissue with planes passing through the nipple (see *Methods, Breast Quadrant Localization*). The quadrant in which the center of mass of the tumor falls defines the breast quadrant localization of the tumor.

### Surgical margin determination

Visualizations of the putative resection volume to be removed during tumor excision can better inform the surgical approach. TumorSight Viz features the ability to display the tumor convex hull surrounding the tumor map, which serves as a general uniform-distance boundary around the tumor. We allowed the distance margin between the tumor and convex hull to be adjustable up to 2 cm, so that the surgeon may consider different surgical margin scenarios. For multifocal tumors, we included the ability to view separate convex hulls around each tumor (a “multicentric” convex hull). This feature is paired with the option to view multifocal tumors with a single convex hull around the entire tumor excision volume (a “unicentric” convex hull), which may aid in deciding between an *en bloc* resection and piecemeal excisions of each tumor nodule.

### Convex hull generation

The TumorSight Viz unicentric convex hull algorithm generates a new tumor mask whose voxel centers are the corners of the original tumor mask, extracting a two-dimensional (2D) triangular surface mesh from the new mask using the marching cubes algorithm in scikit-image, translating the vertices in the mesh along their normal vector by a user-defined margin, and building the convex hull around the translated vertices. The multicentric convex hull algorithm applies the same procedure, albeit separately to each non-contiguous tumor region identified by the scipy.ndimage’s label method. After the hulls are generated, the algorithm checks for overlapping hulls by testing whether each vertex in each hull lies within any other hull. The procedure is applied to all pairs of convex hulls, and all overlapping convex hulls are merged. (See *Methods, Unicentric & Multicentric Convex Hulls* for additional details).

### Radiologist review process establishing ground-truth in the validation set

The 100 cases in the validation dataset were reviewed by 2 breast-specialized radiologists (R1 and R2) for the purpose of selecting quality annotations for ground-truthing (Fig. 2). R1 approved 67 of 91 (73.6%) tumor annotations, while R2 approved 84 of 96 (87.5%). Of the 100 cases, 97 cases featured a reading from either R1 or R2. Some cases were not reviewed because of software issues, trouble finding the lesion, or other issues during the review process. Both radiologists reviewed a common set of 90 tumor annotations. For 83 (92%) of these, at least one radiologist believed that the tumor annotation was of acceptable quality. This finding demonstrates that our annotation pipeline generates viable tumor annotations for use by clinical radiologists, in most cases. For 64 (71%) cases in the common set of 90, both radiologists approved of the quality of the annotations (pre-adjudication). Additionally, both radiologists rejected 7 (8%) cases in the common set. The overall concordance between radiologists was 79% and Gwet’s AC1 was 0.7, both of which indicate that there is a modest degree of variability between even expert human radiological readers when assessing clinical DCE-MRI. Our methodology demonstrates comparable efficacy when assessed against these radiologist performance metrics.

To adjudicate the 19 (21%) discordant cases, the radiologists reviewed the MRIs and annotations together. A consensus was reached to approve 10 (53%) cases and reject 9 (47%). Ultimately, 74 (82%) cases in the common set of 90 were approved by both radiologists (post-adjudication).

A similar vetting procedure was carried out for selecting high-quality multi-tissue annotations for ground-truthing. R1 approved 87 of 90 (97%) reviewed annotations, while R2 approved 88 of 93 (95%) reviewed annotations. Both radiologists reviewed a common set of 86 annotations. Of these, all 86 (100%) were approved by at least one radiologist and 80 (93%) were approved by both radiologists. This finding indicates that our annotation pipeline generates good quality multi-tissue labels for most cases. The overall concordance between radiologists was 93% and Gwet’s AC1 was 0.93, both of which indicate high reliability of radiologists in the vetting of multi-tissue labels.

When considering the tumor and multi-tissue annotations together, R1 and R2 approved both segmentations for 70 (78%) and 68 (73%) cases, respectively. A total of 64 (71%) full MRI annotations were approved by both radiologists in the adjudicated dataset.

### Performance validation of TumorSight Viz 3D tumor label maps

**Fig. 3:**
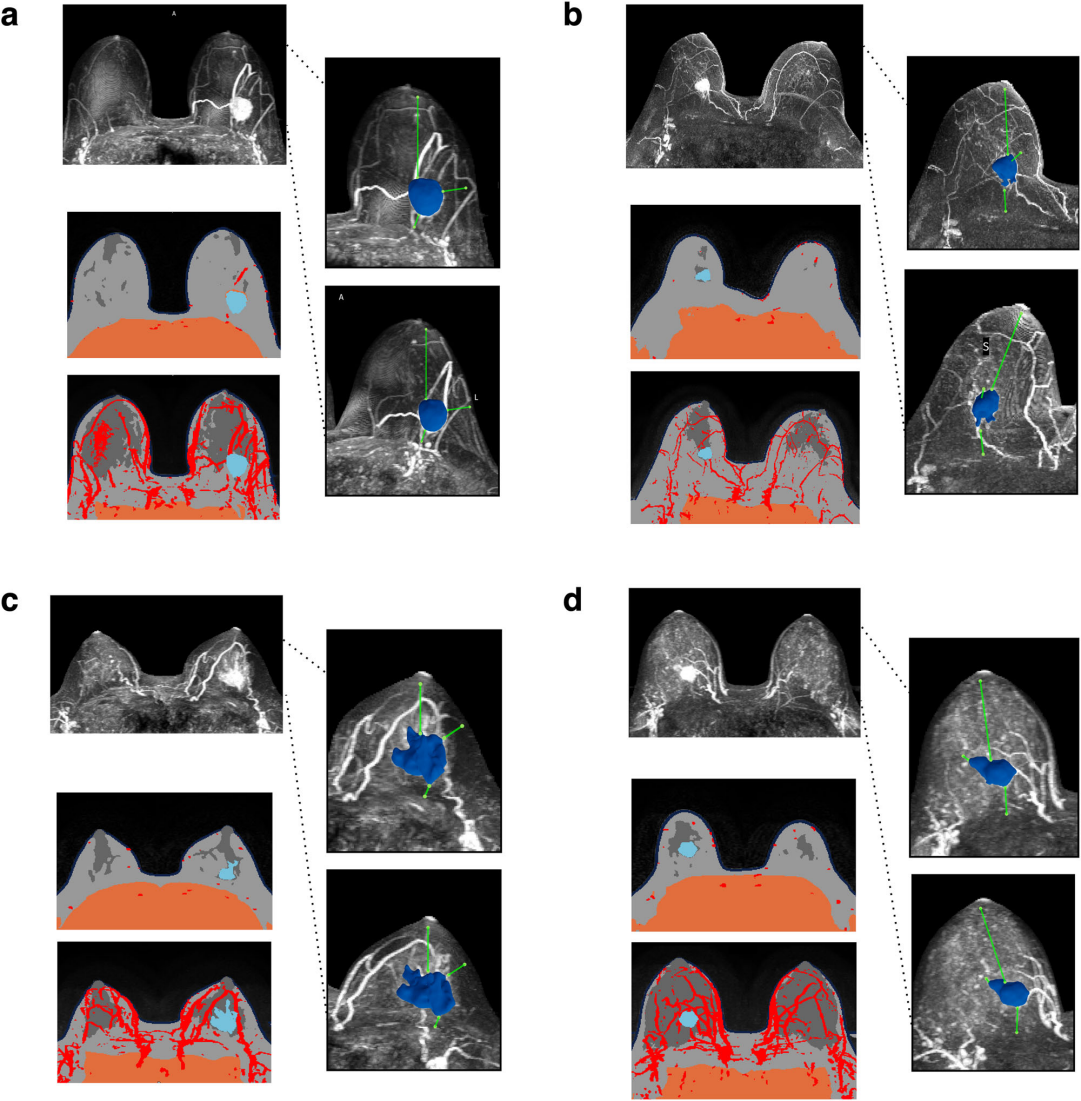
Radiologist-verified depictions of early-stage breast cancer in the UAB validation cohort, as rendered in TumorSight Viz. For each case in (**a**–**d**), the heart-masked DCE-MRI (**left-top**) is shown along with a single MRI slice (**left-mid**) as well as the maximum intensity projection (**left-bottom**). Renderings of TumorSight Viz segmentations from two perspectives are shown at **right**.

**Fig. 4:**
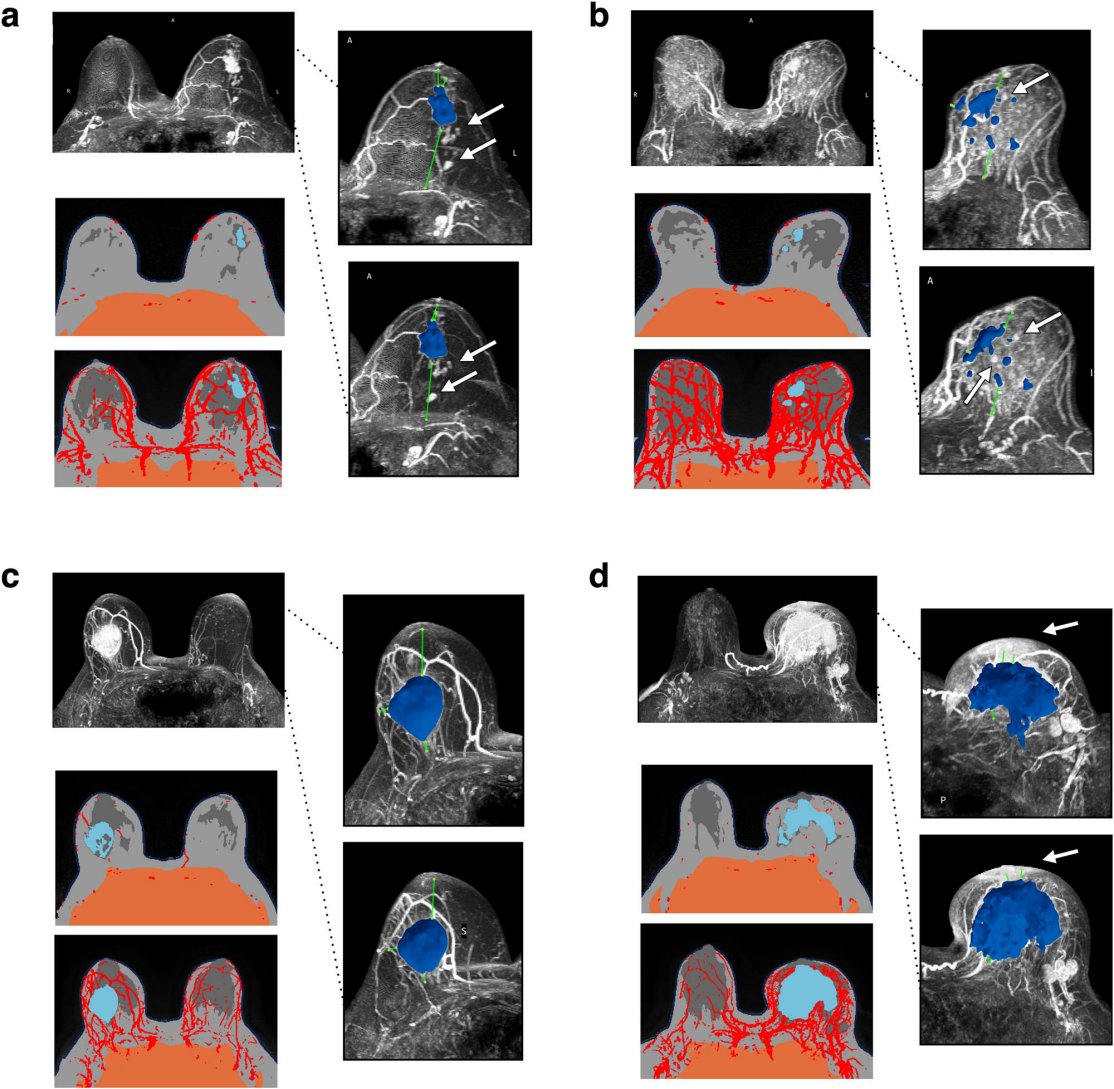
Rejected or adjudicated TumorSight Viz depictions for early-stage breast cancer in the UAB validation cohort. For each case in (**a**–**d**), the heart-masked DCE-MRI (**left-top**) is shown along with a single MRI slice (**left-mid**) as well as the maximum intensity projection (**left-bottom**). Renderings of TumorSight Viz segmentations from two perspectives are shown at right. **a** Renderings of radiologist-rejected segmentations due to under-segmentation. Note several posterior satellite masses (arrow) that were not included in the segmentation. **b** Renderings of radiologist-rejected segmentations due to under-segmentation. Note the multiple distinct tumors (arrows), the type of which were not explicitly included in segmentation model development. **c** Adjudicated case, accepted. Note that the interior of the tumor is only partly segmented, although the overall boundaries of the encompassing tumor are correctly identified. **d** Adjudicated case, rejected. Note the extreme extent of disease presenting into skin and nipple (arrows), which were not accurately segmented by the algorithm.

We provide a compendium of representative images demonstrating overall trends observed in these studies, focusing on both the broad successes of the segmentation model (Fig. 3), as well as areas for improvement (Fig. 4). TumorSight Viz renders clear depictions of these tumor segmentations, which can readily be overlaid on the corresponding MRI data along with segmentations of surrounding tissues.

**Fig. 5:**
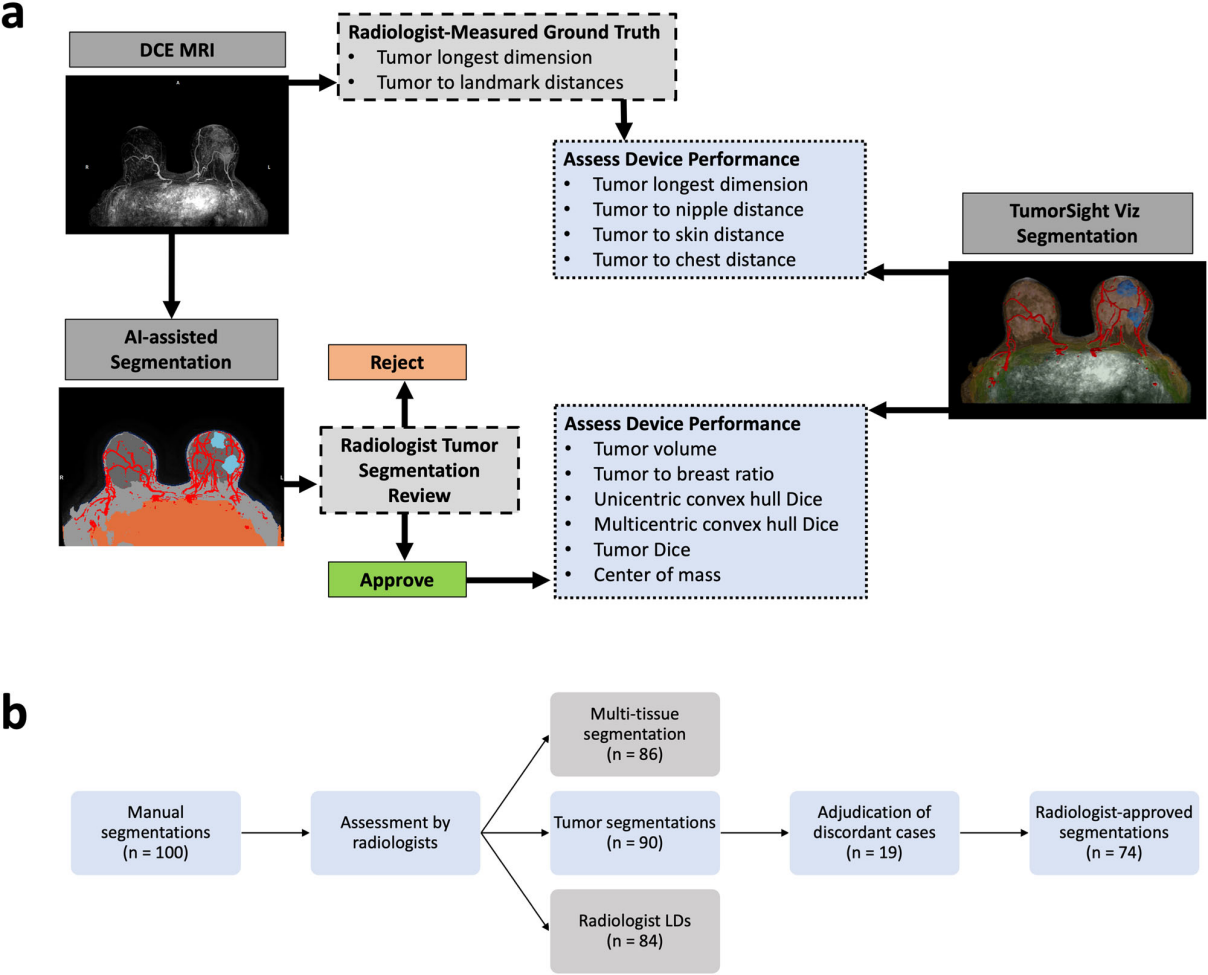
Establishing validation set ground-truth. **a** The overall methodology for ground-truthing and device performance assessment is shown. **b** One hundred tumor and multi-tissue segmentations (AI-assisted manual segmentations) were assessed by 2 radiologists on the following measures: tumor longest dimension (LD) with *n* = 84 cases being completed by both reviewers, tumor segmentation assessment (approve/reject) with *n* = 90 cases being completed by both reviewers, and multi-tissue segmentation assessment (approve/reject) with *n* = 86 cases being completed by both reviewers. A pre-planned adjudication of cases with discordant tumor segmentation calls took place (*n* = 19), resulting in a final set of *n* = 74 radiologist-approved tumor segmentations. This set of segmentations served as the ground-truth comparator in the validation set, when assessing the automatic segmentations generated by TumorSight Viz.

We further assessed the accuracy of TumorSight Viz automatically-generated 3D tumor maps by analysing the degree of overlap with radiologist-approved segmentations (Fig. 5), while also determining the interquartile ranges (IQR) representing the span between the first (25th percentile) and third (75th percentile) quartiles.

The median Sørensen–Dice coefficient (Dice) (see *Methods*) was 75.6% (IQR: 58.6–85.1) for the validation set. For comparison, the median Dice was 85.2% (IQR: 78.1–89.0) for the training set (*n* = 358) and 83.4% (IQR: 74.2–86.2) in the test set (*n* = 46).

**Fig. 6:**
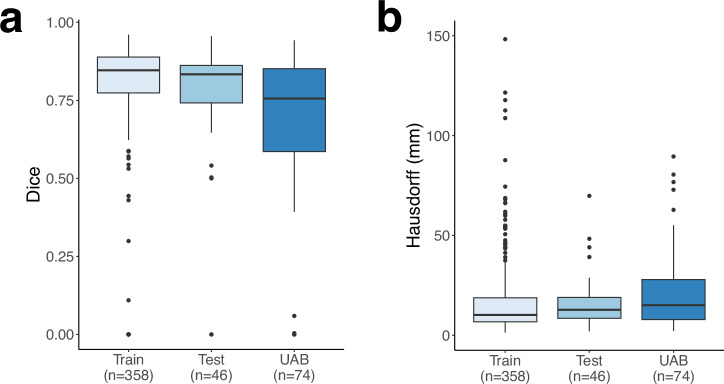
Tumor segmentation metrics. In (**a**), boxplots of the Dice coefficient for the tumor segmentation compared to ground-truth in the training (*n* = 358), testing (*n* = 46), and radiologist-approved UAB (*n* = 74), datasets are shown. The median Dice was 85.2% in the training set (IQR: 78.1–89.0), 83.4% (IQR: 74.2–86.2) in the test set, and 75.6% (IQR: 58.6–85.1) in the radiologist-approved UAB set. In (**b**), the Hausdorff distance is shown for the same datasets. The median Hausdorff distance in the training set was 10.2 mm (IQR: 6.7–19.1), 13.2 mm (IQR: 8.6–26.5) in the test set, and 15.1 mm (IQR: 7.9–27.9) in the radiologist-approved UAB set. Note that one outlier in the train set and two in the test set are not shown in the boxplots, in order to better visualize the central tendencies of the distributions.

In addition to applying Dice as a measure of bulk tumor segmentation quality^31^, we utilized the Hausdorff distance (see *Methods*) as a measure of accuracy in delineating the tumor border^32^. The median Hausdorff distance was 15.1 mm (IQR: 7.8–27.9) for the validation set (Fig. 6). For comparison, the median Hausdorff distance was 10.2 mm (IQR: 6.7–19.1) for the training set (*n* = 358) and 13.2 mm (IQR: 8.6–26.5) for the test set (*n* = 46).

### Performance validation of TumorSight Viz clinical metrics

**Fig. 7:**
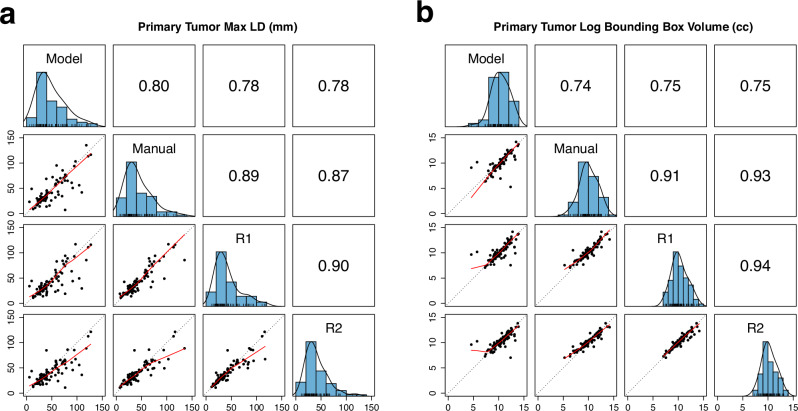
Scatterplot matrix of maximum longest dimension and bounding box volume with Pearson correlation. Along the diagonal, histograms of the maximum longest dimension measurement in (**a**) or log bounding box volume in (**b**) from the model segmentation (Model), manual segmentation (Manual), Radiologist 1 (R1), and Radiologist 2 (R2) are shown. Pairwise comparisons of each case are shown in the scatterplots, with the red lines representing the locally estimated scatterplot smoothing (LOESS) line and the dotted black lines representing perfect agreement. Pearson correlation coefficients are provided above the diagonal.

We assessed the performance of the automated metric determination methods implemented in TumorSight Viz. Summary statistics for automated and manual measurements can be found in Supplementary Table 1. For the longest tumor dimension, TumorSight Viz measurements were linearly related to each radiologist’s direct measurements (Fig. 7). The Pearson correlation coefficients were 0.78 for both radiologists. The correlation between the radiologists was 0.90, indicating significant agreement between radiologists. In addition to evaluating the correlation with ground-truth, we also examined the magnitude of the differences from ground-truth (Supplementary Table 2). The median absolute error in longest tumor dimension between TumorSight Viz and the mean of the 2 radiologists was 6.4 mm (IQR: 2.6–12.4) and 6.3 mm (IQR: 2.8–14.8), respectively. The measurements by radiologists were closer together, with a median difference of 3.2 mm (IQR: 1.6–6.7). These findings demonstrate that TumorSight Viz generally yields suitable metrics for downstream clinical applications.

The log-transformed volumes of TumorSight Viz tumor bounding boxes also showed a linear relationship with computed volumes from direct measurements by radiologists. The correlation coefficients were 0.75 and 0.75 with respect to radiologists R1 and R2, respectively, and 0.94 between radiologists. The median absolute error between TumorSight Viz and radiologist-assigned volumes were 9.5 cm^3^ (IQR: 2.5–50.9) for R1 and 10.7 cm^3^ (IQR: 2.1–42.4) for R2, whereas the median error between the radiologists was 5.6 cm^3^ (IQR: 1.5–22.9).

We also evaluated TumorSight Viz automated measurements of tumor volume, tumor-to-breast-volume ratio, tumor-to-nipple distance, tumor-to-skin distance, tumor-to-chest distance, and the breast quadrant position of the tumor (Supplementary Table 3). The ground-truth for these were extracted from the radiologist-approved segmentations. The median absolute difference was 2.0 cm^3^ (IQR: 0.9–5.7) for tumor volume and 0.002 (IQR: 0.001–0.008) for tumor-to-breast-volume ratio. Distances from tumor to surgical landmarks were also accurate, with median errors of 2.3 mm (IQR: 0.8–8.3) for tumor-to-nipple distance, 1.0 mm (IQR: 0.5–2.8) for tumor-to-skin, and 1.3 mm (IQR: 0.6–3.6) for tumor-to-chest distance. There was 81% concordance on the quadrant localization of the primary tumor. We observed 89% concordance at localize the breast quadrant of the primary tumor.

Similar performance was observed with analyses applied to the extent of disease (Supplementary Tables 1 and 2 and Supplementary Fig. 2).

### Performance validation of TumorSight Viz convex hulls

To assess the accuracy of TumorSight Viz in delineating convex hulls, the Dice score was computed with respect to ground-truth convex hulls generated from radiologist-approved annotations (Supplementary Table 3). The median Dice was 81.5% (IQR: 67.3–90.8), indicating a high degree of overlap between TumorSight Viz and ground-truth convex hulls generated with the unicentric approach. A similar Dice of 81.9% (IQR: 72.0–91.1) was found with a multicentric hull. The results suggest that TumorSight Viz can generate accurate convex hulls around virtual 3D models of both unifocal and multifocal breast tumors.

### Performance validation of TumorSight Viz relative to T stage and cancer subtype

We additionally assessed TumorSight Viz performance relative to breast cancer T stage (T1, T2, T3) (Supplementary Table 4, Supplementary Figs. 3 and 4) and subtype (IDC, ILC, Other) (Supplementary Table 5, Supplementary Figs. 5 and 6). As expected, the observed error generally increased as a function of T stage across all comparisons, such that larger dimension values for later T stages had larger absolute errors. IDC and invasive lobular carcinoma (ILC) showed similar absolute errors, indicating that the segmentation model is robust to distinct pathological presentations of disease, particularly the more diffuse or morphologically complex presentation of ILC^33,34^. In contrast, “Other” cancer types had notably larger errors, likely due to insufficient sampling of these subtypes during model development and the small sample size in the validation set.

### Failure modes for tumor segmentation

We identified the 10 cases with the largest absolute difference between tumor volumes extracted from TumorSight Viz tumor annotations as compared to the radiologist-approved tumor annotations. We found that 9 cases had 1 or more of the following: heterogeneous or large degrees of fibroglandular tissue, moderate or marked background parenchymal enhancement, or non-mass enhancement. 7 cases had 2 or more from the above list. When looking at the 10 cases with the smallest absolute differences in tumor volume, we found that only 2 cases showed a single issue: heterogenous fibroglandular tissue. None had moderate or marked background parenchymal enhancement or non-mass enhancement. These results indicate the specific areas in which our model requires further refinement, helping to direct future software development.

## Discussion

TumorSight Viz is a medical device software platform that assists in clinical decision-making through detailed 3D renderings of tumors in their anatomic context. The core technology employs a computer vision model developed on a large dataset of de-identified breast cancer patient DCE-MRIs. TumorSight Viz uses a deep-learning model that segments breast tumors and surrounding anatomy on DCE-MRIs to render 3D visualizations (Fig. 1)^27^. While others have previously developed 3D breast tumor segmentation models^17,35–40^, the TumorSight Viz platform offers several benefits. TumorSight Viz segments and visually maps 5 additional tissue types, providing surgeons with a “lay of the land” of the tumor with key anatomical landmarks (Fig. 1, right-top). Multi-tissue segmentation provides 3D visualizations of a tumor and its context within the body, and also enable automated quantitative assessments including tumor-to-landmark distances and breast quadrant localization (Fig. 1, right-mid). The observed level of precision in the platform’s measurements is on the same scale as the spatial resolution of MRI, which typically ranges from 0.7 to 1.6 mm depending on scanner type^41,42^, indicating that device performance meets resolution standards for surgical precision that are inherent to clinical MRI data. The results support the relevance of TumorSight Viz generated measurements compared to real-world surgical implementation.

TumorSight Viz expands upon the existing shortlist of software platforms for DCE-MRI visualization, including DynaCAD and CADstream^43,44^. TumorSight Viz represents the first stage of a broader effort to incorporate the wealth of patient data and emerging treatment response forecasting into a therapeutic pipeline customized on a per patient basis. The platform integrates with a suite of precision cancer analysis software, spanning from disease diagnosis, drug response forecasting and dose optimization, clinical and surgical decisional support, and long-term disease recurrence prediction. TumorSight Viz therefore represents an initial phase in establishing this broader end-to-end treatment platform for patients.

The workflow enabled by TumorSight Viz supports broad medical initiatives such as Cancer Moonshot, which aim to enhance personalized cancer treatment and intervention through an expanded toolkit of precision medicine techniques and tools. TumorSight Viz incorporates surgical clinical decision support functionalities to help visualize hypothetical resection margins, as represented by convex hulls around the 3D tumor map (Fig. 1, right-bottom). Such guiding margins can form a foundational surgical guideline which demarcate the minimal boundary for tumor removal. The convex hull can be expanded to increase the boundary of tissue removal to minimize risk of recurrence depending on the margin of safety required and the likelihood of achieving desired cosmetic outcomes. Several risk factors for positive surgical margins have been identified^45–47^, offering objective avenues for adjusting both pre- and intra-operative imaging and surgical procedures.

Recent studies have suggested the safety of oncoplastic BCS for selected women with multifocal breast tumors^48^. As such, the ability of TumorSight Viz to depict separate convex hulls for tumor foci, or a single convex hull around the entire tumor, may aid in deciding between an *en bloc* resection or separate excisions of each tumor focus. We found that convex hulls generated by TumorSight Viz were comparable to those generated from radiologist-approved tumor segmentations. Additional validation is needed, but the early assessment of TumorSight Viz to approximate surgical margins is promising.

The ability to rapidly assess and annotate these 3D label maps from routine standard of care medical imaging obtained for breast cancer patients must accurately reflect the breast tumor(s) and their local anatomy (Fig. 1, left), and it is paramount that these depictions faithfully reflect radiological interpretation of the patient’s true anatomy (Fig. 1, right-top). In the current study, we found a high degree of agreement between the ground-truth radiologist image assessment and the automatic TumorSight Viz breast segmentation assessment, as shown by 2 complimentary evaluation metrics. Dice analysis revealed significant volumetric overlap of bulk tumor segmentations between human readers and the segmentation model. Additionally, the Hausdorff distance demonstrated that tumor borders were accurately delineated, in the 1–3 mm range. These results align with findings demonstrating that 2 mm margins minimize degrees of recurrence^49^, suggesting a suitable range for surgical performance. Accurate demarcation of tumor borders is particularly relevant for assigning surgical margins and gauging chest wall or skin invasion for staging purposes. TumorSight Viz performance metrics demonstrate the utility of TumorSight Viz in facilitating these crucial aspects of surgical planning.

A widely-applicable surgical clinical decision support tool should generalize across institutions with varying scanners and imaging protocols in order to facilitate deployment across numerous institutions and clinical settings. The validation dataset used in this study was derived from an institution that did not contribute to the training and testing datasets. As expected, a modest reduction in performance was observed with the use of data from an unseen institution, a practical reality in nearly all machine learning studies. However, given the strong performance of the model on a distinct dataset, this validation study ultimately demonstrates the segmentation model’s broad generalizability for 3D tumor renderings and analysis. A major strength of our study is our use of full 3D tumor segmentations to validate our virtual tumor model. Alternative methods that rely on 2D slices, maximum intensity projections, or regions of interest have been used, but these approaches suffer from information loss relative to 3D annotations^36,50,51^. The TumorSight Viz methodology extracts morphological features within a 3D spatial lattice, rather than interpolating detail from 2D imaging, thereby enhancing informed treatment planning efforts.

Analysis of radiological features (“radiomics”) in medical imaging is a rapidly-expanding field, with the overarching goal of using image properties as the basis for disease status analysis and therapy response prediction^52–54^. TumorSight Viz allows researchers to explore the radiological and radiomic features of breast tissues and tumors as a starting point for disease diagnostics and clinical decision support. The automated 3D tissue map further allows for extraction of radiomic features with minimal labor, enabling researchers to use these in downstream applications.

TumorSight Viz was designed to better enable economical cancer surgical decision-making, thus helping to combat the continued rise in patient surgical costs despite a lack of commensurate reimbursement^55^. Hospitals are expected to shift towards solutions that enable surgeons to make better and more precise decisions pre-operatively, with the goal of guiding better anticipation of treatment outcomes. TumorSight Viz is poised to help expedite these surgical efforts, therefore holding promise for reducing uncertainty, improving patient care decisions, and reducing costs. The platform’s extensive visualization and decisional support tools minimize intra-operative decisions, shifting this planning to informed discussions between patients and physicians during the consultation phase.

The multi-institutional nature of the segmentation model’s training and testing sets provides the basis for broad generalizability of TumorSight Viz performance. However, there are notable limitations inherent to our training data. Our study cohort was limited to subjects eligible for neoadjuvant chemotherapy. Heterogenous or extreme fibroglandular tissue, moderate or marked background parenchymal enhancement, and non-mass enhancement (all known radiological challenges when reading MRI) were particularly associated with errors in tumor volume. We note that the model training and testing sets were biased toward cT2 tumors (~60%), whereas the UAB cohort was biased toward cT3 tumors (~70%). This discrepancy between the tumor types used in model training and subsequent application in this study may have resulted in some loss of performance on the UAB cohort. However, given the overall strong performance of the model on a distinct dataset, this validation study demonstrates the segmentation model’s broad applicability in 3D tumor renderings and analysis. Future multi-institutional prospective studies aimed at extensively assessing the generalizability and broader utility of TumorSight Viz, as well as efforts piloting clinical implementation, are ongoing.

The TumorSight Viz platform is designed to aid surgeons in planning their approach in breast cancer treatment. As a step towards the goal of comprehensive surgical-planning support, TumorSight Viz features precision 3D renderings of tumors in the context of key anatomical structures, quantitative measures of tumor morphology and tumor-landmark relationships, as well as visual approximations of resection margins. Many of the functionalities introduced in this study are derived from the platform’s multi-tissue 3D label map that encompasses the tumor and all clinically relevant tissues. Enhanced qualitative and quantitative capabilities for assessing surgical approach and decision-making requires high performance in accurately labeling all key anatomical structures. TumorSight Viz offers to streamline surgical procedures, and thus enable surgeons to better assess surgical treatment options and facilitate outcomes tailored for every patient and their corresponding unique anatomy. TumorSight Viz will continue to be updated and expanded as development proceeds for other indications and organ systems.

## Methods

### Ethics approval and consent to participate

De-identified patient data from the University of Alabama (UAB) were used for this study, for which an IRB waiver was granted. The investigators complied with all relevant ethical regulations and those specified under the Declaration of Helsinki.

### Validation dataset annotations

For all cases, ground-truth tumor labels were generated using a hybrid AI-human approach. We first automatically generated preliminary tumor labels using an AI-powered labeling tool^27^. These labels were then edited, when necessary, by trained laypeople to better mask the tumor on raw MRIs. Fully-labeled DCE-MRIs were produced by merging the tumor labels (i.e., as derived from verified automatic tumor segmentations or trained layperson annotators) with the automatic multi-tissue labels generated by TumorSight Viz. We refer to these as “AI-assisted tumor and multi-tissue labels” throughout this manuscript.

### Radiologist-approved ground-truth annotation

To ensure performance of TumorSight Viz was validated against clinical standards, ground-truth labels were reviewed by 2 board-certified radiologists. Both radiologists are fellowship-trained in breast imaging and have 8 years of experience each. The workflow for ground-truth generation in this study is shown in Fig. 2.

To prevent biasing of reader review, radiologists made measurements of tumor dimensions on raw DCE-MRI data prior to being shown either AI-assisted or automatic TumorSight Viz segmentations/annotations. After measurement determination, radiologists were then asked to review AI-assisted segmentations and mark these as accept/reject for both the tumor and multi-tissue segmentations. The multi-tissue segmentation was considered as a unit, such that the entirety of the 5 tissue segmentations were either accepted or rejected in their totality. The tumor approve/reject and multi-tissue approve/reject decisions were not linked or otherwise dependent on one another. Radiologists were additionally asked to review automatic segmentations of the tumor as generated by TumorSight Viz. They were instructed to base their decision on whether the tumor regions were annotated completely and exclusively. Cases with discordant ratings were adjudicated at a consensus conference between the radiologists. Only tumor annotations that were ultimately approved by *both* radiologists were used for ground-truthing in this study. Radiologists were asked to approve/reject TumorSight Viz segmentations, but did not manually modify the segmentations.

A similar procedure was carried out for vetting multi-tissue annotations. Radiologists were instructed to consider under- or over-segmentation of fibroglandular and adipose tissue, unsegmented major vessels, and patches of unsegmented skin in their review of the multi-tissue segmentation before ultimately accepting or rejecting it. There was no adjudication process for discordant reviews of multi-tissue annotations.

We found a high degree of agreement between the automatic measurements on approved segmentations and direct measurements on MRI (See *Results – Performance Validation*). We proceeded to use the radiologist-approved annotations as ground-truth measurements, allowing us to compute ground-truth tumor volume, tumor-to-breast-volume ratio, tumor localization, tumor-to-nipple distance, tumor-to-skin distance, and tumor-to chest distance.

Similarly, ground-truth unicentric and multicentric convex hulls were generated using radiologist-approved tumor annotations.

### Breast box construction

The breast box was constructed using 5 key points that define the boundaries of the box. We initially identified the center of mass of the segmented breast tissues and the most anterior point along the image midline. Using these, we defined a new point that is a fixed distance (6 mm) posterior to the anterior point and aligned with the center of mass in the medial-lateral direction. We then found the 2 points on the surface of the labeled skin that are closest to the 6 mm fixed-distance point in both the transverse and sagittal projections (4 points total). The remaining fifth point was determined as the most anterior point on the surface of the skin. These 5 points define an initial guess for the extent of the breast in the horizontal, longitudinal, and anterior directions.

Next, checks were made to ensure no segmented breast tissue was excluded from the box. For each of the 5 points, if the corresponding box plane intersects breast tissue, the point is moved along the skin boundary in the direction that increases the box volume until no intersection occurs. As an example, although a point within the inframammary crease may define the inferior extent of the initial bounding box, a small portion of the breast tissue may actually be inferior to it. By iteratively moving the point along the surface of the skin in the inferior direction, we can adjust its location to a point slightly below the crease such that no more breast tissue is excluded. Once all these corrections are made, the extents of the final box in each direction are defined.

### Breast quadrant localization

Breast quadrants were computed using the nipple location, the breast box, and the tumor segmentation. Initially, the breast was split into 4 quadrants using the transverse and sagittal planes. However, by allowing the sagittal plane to rotate slightly around the superior-inferior axis, we obtained better quadrant agreement with our clinical data. The rotation was done by finding the rotation angle that results in equal amounts of breast tissue (defined as tissue within the breast box) on each side of the plane. Additionally, the plane is defined to always pass through the nipple. Once the breast is split into the 4 quadrants, the quadrant containing the tumor center of mass is identified, and the fraction of tumor volume in each quadrant is determined.

### Unicentric Hulls

To compute the convex hull around a tumor, we utilize scipy.spatial’s ConvexHull method. This method takes a set of points (an (N, 3) array of 3D point coordinates) and creates a convex hull object defined by vertices, facets, and neighbor information.

The points for which we create the convex hull are not the set of points defining the tumor volume, but are instead the points defining the tumor surface. This surface is constructed using skimage.measure’s marching_cubes method, which provides attributes for the vertices and surface normals. This allows us to find the convex hull of the tumor plus a surgical margin, by letting us first expand the tumor surface by moving its vertices away from the tumor surface (along the surface normals) and then computing the convex hull of these new surface vertex positions.

### Multicentric Hulls

Although we can treat all tumor voxels as a single set of points and compute their “unicentric” convex hull, in cases where the tumor is made up of multiple, disconnected regions, we might be more interested in the spatial distribution of their individual convex hulls. We call this the “multicentric” tumor convex hull.

We first cluster the tumor voxels using scipy.ndimage’s label method. This uses a given structure element to find clusters of 1 s in a binary mask. This algorithm works by finding isolated regions of tumors voxels that do not have any neighboring tumor voxels (i.e., even a one-voxel thick layer of separation will result in two separate regions being identified). The algorithm gives each cluster a unique label and makes it possible to analyze each cluster of points individually. We then compute the convex hull of each cluster.

In cases in which adding a surgical margin to each cluster’s expanded surface causes them to overlap, we treat the overlapping surfaces as one and compute their combined hull (with the chosen margin). Overlapping surfaces are determined by checking if any of the vertices in one convex hull lie within another.

### Evaluation of tumorsight viz 3D label maps, clinical metrics, and Convex Hulls

To assess the quality of TumorSight Viz tumor segmentations with respect to ground-truth segmentations, the following 2 complimentary evaluation metrics were computed. First, we utilized the Sørensen–Dice coefficient (Dice), which is widely used in the computer vision literature to gauge the similarity of 2 segmentations and is defined as:

1 $${Dice}=\frac{2{TP}}{2{TP}+{FP}+{FN}}$$

where TP, FP, and FN are the pixel-wise numbers of true positives, false positives, and false negatives, respectively. Second, we used the Hausdorff distance d_H_, which is defined as:

2 $${d}_{H}(X,\,Y)=\max \left\{{su}{p}_{x\in X}{\rm{d}}\left(x,Y\right),{su}{p}_{y\in Y}{\rm{d}}\left(X,y\right)\right\}$$

where $d\left(a,B\right)=\,\mathop{\inf }\limits_{b\in B}{d}\left(a,b\right)$, $d$ is a distance measure from point $a\in X$ to subset $B\subseteq X$, and $X$ and $Y$ are tumor boundary pixels in the TumorSight Viz and ground-truth segmentations, respectively. It is a measure of how far apart 2 sets are from each other and has been previously used to evaluate the performance of segmentation networks in accurately delineating tumor boundaries^56^.

The accuracies of TumorSight Viz tumor dimension and bounding box volume measurements were assessed using the intra-class correlation, Pearson correlation, absolute error, and relative error with respect to each radiologist’s direct measurements. These statistics were also computed between the 2 radiologist’s measurements to serve as benchmarks for evaluating TumorSight performance. Similar assessments were performed to estimate the agreement between measurements by radiologists and measurements extracted from radiologist-approved annotations to affirm the quality of the annotations.

The accuracy of TumorSight Viz measurements of tumor volume, tumor-to-breast-volume ratio, tumor-to-nipple distance, tumor-to-skin distance, and tumor-to chest distance was assessed using the signed, absolute, and relative errors compared to ground-truth segmentations. Performance in breast quadrant localization was quantified using concordance, and the accuracy of convex hulls was evaluated using the Dice score. In each case, the reference was provided by the corresponding metric extracted from radiologist-approved segmentations. Interquartile ranges (IQR) representing the span between the first (25^th^ percentile) and third (75^th^ percentile) quartiles were also calculated.

### Sample size determination and acceptance criteria

The percentage of cases approved by radiologists was calculated to assess the performance of our internal AI-assisted annotation pipeline for tumor and multi-tissue segmentations. Based on an early pilot study, a 65% approval rate was considered acceptable. In order to exclude 65% from the lower bound of a one-sided exact confidence interval (CI) of the approval rate of segmentations, while assuming a true approval rate of 75% or higher, a sample size of at least 88 cases was required. To account for anticipated missing reviews (due to issues with software, trouble finding the lesion, etc.), we arrived at a final sample size of 100 cases.

The concordance and Gwet’s AC1 between radiologist approval/rejection calls was calculated to evaluate the reliability of radiologists at vetting ground-truth for both the tumor and multi-tissue segmentations.

## Supplementary information

Supplementary Material

## Data Availability

The datasets generated during and/or analysed during the current study are presented in the main body text as well as the Supplementary information.

## Code availability

Statistical methods implemented in TumorSight Viz are described in the main body text. The code for the TumorSight Viz platform is the intellectual property of SimBioSys, Inc.
