# Supplementary material for: Performance of an AI-powered visualization software platform for precision surgery in breast cancer patients
Source: NPJ Breast Cancer. 2024 Nov 14;10:98. doi: 10.1038/s41523-024-00696-6 (PMC11564706; doi:10.1038/s41523-024-00696-6)
Supplement: Supplementary file 1 — Supplementary Material [file 41523_2024_696_MOESM1_ESM.pdf]

SUPPLEMENTAL INFORMATION

TITLE

Performance of an AI-powered Visualization Software Platform for Precision Surgery in Breast Cancer Patients

AUTHORS

Michelle Weitz[1], JR Pfeiffer[1], Snehal Patel[1], Matthew Biancalana[1], Arda Pekis[1], Vignesh Kannan[1], Evandros Kaklamanos[1], Amanda Parker[1], Jesse Bucksot[1], José Rubio Romera[1], Ryan Alvin[1], Yuhan Zhang[1], Andrew T Stefka[1], Dorys Lopez-Ramos[1], Joseph R Peterson[1], Anuja K Antony[1], Kathryn Zamora[2], Stefanie Woodard[2]

AFFILIATIONS

1 SimBioSys, Inc. Chicago, IL.

2 University of Alabama at Birmingham School of Medicine, Department of Radiology. Birmingham, AL.

SUPPLEMENTAL FIGURES + TABLES

A

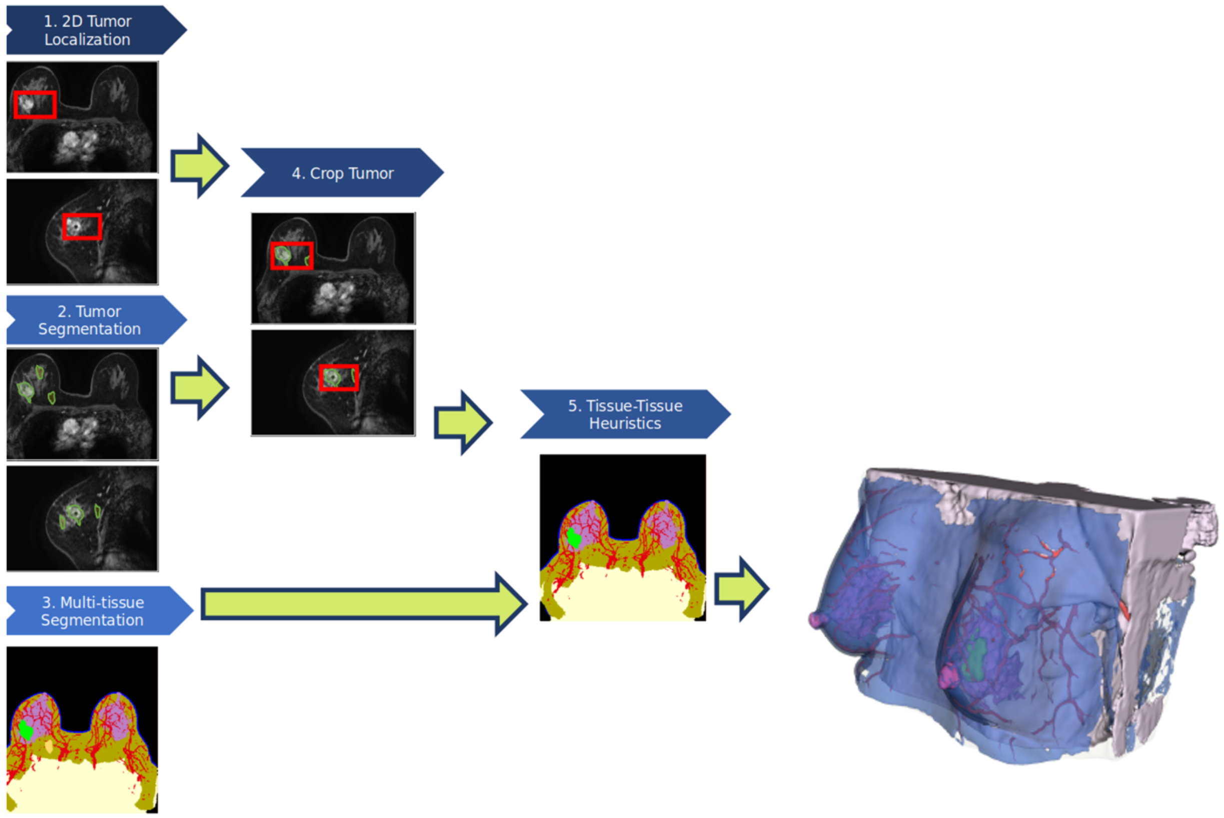

B

Tumor Dataset	Patients
Training	358
Validation	44
Testing	46
Hold-out	48

C

Multi-Tissue Dataset	Patients
Training	50
Validation	15
Hold-out	32

Supplemental Figure 1: Overview of the tumor and multi-tissue segmentation model. (A) Schematic for segmentation pipeline, as well as the how separate datasets are integrated to form the overall tumor + multi-tissue segmentations. (B) Training, validation, testing, and hold-out datasets for the tumor and (C) multi-tissue segmentation models.

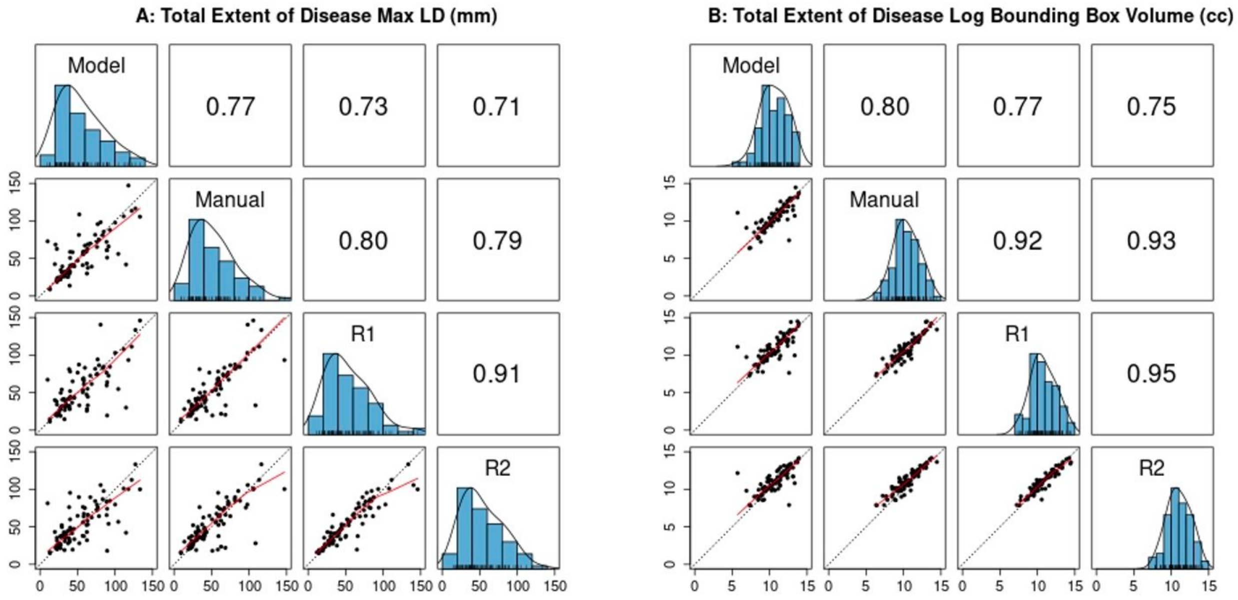

Supplemental Figure 2: Scatterplot matrix of maximum longest dimension and bounding box volume with Pearson correlation. Along the diagonal, histograms of the maximum longest dimension measurement (A) or log bounding box volume (B) from the model segmentation (Model), manual segmentation (Manual), Radiologist 1 (R1), and Radiologist 2 (R2) are shown. Pairwise comparisons of each case are shown in the scatterplots, with the red lines representing the locally estimated scatterplot smoothing (LOESS) line and the dotted black lines representing perfect agreement. Above the diagonal Pearson correlation coefficients are provided.

Supplemental Table 1: Longest dimension (LD) and bounding box volume summary statistics

**Primary Tumor**						
**Maximum LD (mm)**	Min.	25th %ile	Median	Mean	75th %ile	Max.
Model segmentation	5.75	27.98	39.69	48.74	63.35	127.36
Manual segmentation	7.17	25.37	35.23	43.53	58.81	135.18
Reviewer 1	11.05	25.01	34.27	42.55	52.10	117.01
Reviewer 2	11.00	26.14	35.58	40.94	50.69	121.42
**Bounding box volume (cm^3)**	Min.	25th %ile	Median	Mean	75th %ile	Max.
Model segmentation	0.10	9.41	30.21	111.42	119.61	1,101.59
Manual segmentation	0.19	8.26	19.11	92.46	79.20	1,433.18
Reviewer 1	1.17	10.35	22.69	104.74	69.79	1,420.87
Reviewer 2	1.12	10.67	22.43	76.95	75.12	1,024.22
**Total Extent of Disease**						
**Maximum LD (mm)**	Min.	25th %ile	Median	Mean	75th %ile	Max.
Model segmentation	10.10	31.77	45.39	54.60	75.33	133.25
Manual segmentation	8.89	30.01	42.79	52.18	68.04	147.13
Reviewer 1	11.51	31.73	44.40	53.88	73.88	146.10
Reviewer 2	15.03	33.21	46.94	53.73	74.81	133.27
**Bounding box volume (cm^3)**	Min.	25th %ile	Median	Mean	75th %ile	Max.
Model segmentation	0.30	11.07	39.99	143.07	158.00	1,096.65
Manual segmentation	0.57	10.82	26.62	124.56	109.59	1,895.62
Reviewer 1	1.40	16.13	39.63	186.37	165.53	1,959.62
Reviewer 2	2.57	20.93	57.84	168.49	189.45	1,420.87

Supplemental Table 1 Legend: Summary statistics for the tumor longest dimension (LD) and bounding box volume from the primary tumor and total extent of disease are shown in the table. The bounding box volume was measured by taking the axis-aligned LD measurements in all three

planes and creating a box. In the top half of the table, summary statistics from the maximum LD (mm) and bounding box volume (cm^3) are given for the model segmentations, manual segmentations, Reviewer 1 and Reviewer 2. In the bottom half of the table, these same metrics are given for the total extent of disease (primary tumor plus the totality of the extent of cancerous disease in the breast).

Supplemental Table 2: Primary tumor and total extent of disease error metrics

Primary Tumor Maximum LD					
Source		Absolute Error (mm)		Relative Error (%)	
Meas. 1	Meas. 2	Mean (SD)	Median (Range)	Mean (SD)	Median (Range)
Reviewer 1	Reviewer 2	6.54 (9.20)	3.15 (0.27, 49.70)	3.42 (23.31)	-1.80 (-50.41, 73.83)
Model	Manual	10.96 (14.20)	4.59 (0.12, 69.31)	29.78 (113.90)	8.94 (-79.31, 966.07)
Model	Reviewer 1	11.46 (14.55)	6.40 (0.05, 80.00)	24.29 (59.17)	9.62 (-83.10, 302.00)
Model	Reviewer 2	12.00 (14.66)	6.27 (0.04, 73.35)	26.76 (76.20)	10.63 (-81.78, 595.33)
Manual	Reviewer 1	7.39 (9.39)	4.04 (0.38, 49.54)	4.26 (31.19)	0.71 (-62.99, 114.44)
Manual	Reviewer 2	8.30 (10.40)	4.85 (0.16, 48.79)	4.99 (29.46)	-1.80 (-57.55, 93.29)
**Primary Tumor Bounding Box**					
**Source**		**Absolute Error (cm^3)**		**Relative Error (%)**	
**Meas. 1**	**Meas. 2**	**Mean (SD)**	**Median (Range)**	**Mean (SD)**	**Median (Range)**
Reviewer 1	Reviewer 2	47.95 (150.76)	5.63 (0.02, 1,201.06)	26.35 (106.18)	-9.48 (-69.74, 546.41)
Model	Manual	50.93 (124.87)	8.33 (0.03, 749.57)	1,557.35 (13,170.26)	22.83 (-98.90, 11,9320.72)
Model	Reviewer 1	64.69 (155.72)	9.48 (0.06, 1,080.18)	222.19 (1,360.21)	9.58 (-99.39, 12,008.52)
Model	Reviewer 2	51.36 (110.30)	10.72 (0.01, 584.25)	306.13 (2,263.42)	1.65 (-99.35, 20,463.85)

Manual	Reviewer 1	52.68 (177.80)	8.57 (0.03, 1,134.53)	2.68 (89.63)	-15.49 (-97.21, 393.96)
Manual	Reviewer 2	42.71 (123.40)	8.64 (0.23, 963.83)	5.94 (100.64)	-19.74 (-89.84, 100.64)
**Total Extent of Disease Maximum LD**					
**Source**		**Absolute Error (mm)**		**Relative Error (%)**	
**Meas. 1**	**Meas. 2**	**Mean (SD)**	**Median (Range)**	**Mean (SD)**	**Median (Range)**
Reviewer 1	Reviewer 2	8.28 (9.46)	4.73 (0.22, 46.08)	1.41 (32.95)	-5.13 (-50.03, 231.63)
Model	Manual	12.54 (15.46)	5.13 (0.12, 72.83)	12.28 (41.67)	5.92 (-86.11, 188.40)
Model	Reviewer 1	14.18 (16.71)	7.95 (0.17, 84.67)	12.21 (62.38)	1.24 (-84.96, 358.57)
Model	Reviewer 2	14.54 (15.77)	9.37 (1.09, 72.57)	9.29 (58.62)	-4.11 (-87.58, 395.23)
Manual	Reviewer 1	11.15 (14.40)	5.48 (0.10, 75.52)	4.92 (52.96)	-5.55 (-58.25, 234.07)
Manual	Reviewer 2	11.75 (13.43)	7.49 (0.21, 80.48)	3.81 (57.46)	-9.36 (-55.48, 286.70)
**Total Extent of Disease Bounding Box**					
**Source**		**Absolute Error (cm^3)**		**Relative Error (%)**	
**Meas. 1**	**Meas. 2**	**Mean (SD)**	**Median (Range)**	**Mean (SD)**	**Median (Range)**
Reviewer 1	Reviewer 2	69.00 (182.66)	17.62 (0.22, 1,158.22)	0.02 (86.57)	-22.29 (-73.82, 603.53)
Model	Manual	69.50 (163.87)	12.72 (0.03, 1,188.55)	282.98 (1,964.30)	22.43 (-99.53, 17,772.01)
Model	Reviewer 1	100.49 (217.33)	19.24 (0.06, 1,446.06)	214.36 (1,609.12)	-15.88 (-99.54, 14,292.36)

Model	Reviewer 2	82.71 (116.86)	25.72 (0.66, 578.44)	133.66 (1,197.58)	-31.62 (-99.84, 10,760.25)
Manual	Reviewer 1	97.87 (263.59)	21.18 (0.25, 1,459.50)	-14.09 (76.06)	-32.31 (-90.00, 370.72)
Manual	Reviewer 2	75.33 (145.22)	24.59 (0.39, 1,057.60)	-31.87 (50.61)	-39.83 (-87.64, 201.72)

Supplemental Table 2 Legend: Absolute and relative error metrics are given for pairwise comparisons between the model segmentations, manual segmentations, Reviewer 1, and Reviewer 2 on longest dimension (LD) and bounding box volume for the primary tumor and total extent of disease.

Supplemental Table 3: Clinical Measurement Error Metrics

Signed Difference						
	Min.	25th %ile	Median	Mean	75th %ile	Max.
Tumor Volume (cm^3)	-34.33	0.44	1.57	4.61	4.56	62.81
Tumor-to-Breast-Volume Ratio	-0.02	0.0004	0.002	0.005	0.004	0.09
Tumor-to-Nipple Distance (mm)	-28.59	-3.09	-0.23	-0.61	1.35	33.20
Tumor-to-Skin Distance (mm)	-16.70	-1.22	-0.43	-0.50	0.53	13.18
Tumor-to-Chest Distance (mm)	-30.72	-1.89	-0.70	-1.01	0.00	15.49
**Absolute Difference**						
Tumor Volume (cm^3)	0.11	0.90	1.96	7.06	5.66	62.81
Tumor-to-Breast-Volume Ratio	0.0001	0.001	0.002	0.007	0.008	0.09
Tumor-to-Nipple Distance (mm)	0	0.81	2.31	6.33	8.28	33.20
Tumor-to-Skin Distance (mm)	0	0.45	1.03	2.33	2.78	16.70
Tumor-to-Chest Distance (mm)	0	0.57	1.25	3.07	3.60	30.72
**Relative Percent Difference (%)**						
Tumor Volume	1.25	15.74	28.98	45.08	61.61	198.94
Tumor-to-Breast-Volume Ratio	1.25	15.74	28.98	45.08	61.61	198.94

Tumor-to-Nipple Distance	0	2.28	6.48	27.15	40.01	200.00
Tumor-to-Skin Distance	0	7.42	19.87	50.41	53.50	200.00
Tumor-to-Chest Distance	0	4.46	15.04	36.99	39.40	200.00
**Dice**						
Unicentric Hull Dice	0%	67.3%	81.5%	74.1%	90.8%	96.2%
Multicentric Hull Dice	0%	72.0%	81.9%	75.1%	91.1%	96.2%

Supplemental Table 3 Legend: Clinical measurement error metrics for the 64 cases with radiologist-approved tumor and multi-tissue segmentations are shown in the table. For signed differences, the difference between the model segmentations and radiologist-approved manual segmentations was taken, so that a negative number indicates the model under-segmented or under-measured compared to the radiologist-approved manual segmentations and a positive number indicates that the model over-segmented or over-measured compared to the radiologist-approved manual segmentations.

Performance by T Stage

We examined the longest dimension (LD) measurement error between the radiologists (R1 and R2), the manually verified AI-assisted annotations (Manual), and the TumorSight Viz segmentations (Model) stratified by T stage.

Of the 97 cases with radiologist measurements, 26 (27%) have ground-truth T stage from diagnostic data provided by UAB and 71 (73%) do not have such data. For the purpose of this analysis, we use the diagnostic T stage data where available, and calculate it based on the average of the two radiologists' measurements when unavailable.

Of the 97 cases with radiologist measurements, 82 have primary tumor measurements from both radiologists as well as from the TumorSight Viz segmentation. Of these 82, 13 (16%) are T1, 48 (59%) are T2, 20 (24%) are T3, and 1 (1%) is T4. Due to the small T4 sample size and because the single patient qualifies as a T3 based on the LD measurement alone, the T4 case is grouped with the T3 cases. Absolute error and relative percent difference from pairwise comparisons of all measurement sources can be found in Supplemental Table 4 and are shown in Supplemental Figures 3 and 4.

Supplemental Table 4: Pairwise Measurement Error of Primary Tumor Maximum LD by T Stage

Primary Tumor Maximum LD					
Source		Absolute Error (mm)		Relative Percent Difference (%)	
Meas. 1	Meas. 2	Mean (SD)	Median (Range)	Mean (SD)	Median (Range)
T1 (N = 13)					
R1	R2	2.59 (2.32)	1.97 (0.53-8.03)	14.40 (13.89)	11.40 (3.17-53.46)
Model	Manual	10.51 (18.72)	3.99 (2.36-69.31)	37.90 (45.15)	21.25 (8.24-165.70)
Model	R1	10.37 (15.82)	3.96 (0.38-57.46)	34.44 (37.08)	20.52 (1.93-120.32)
Model	R2	11.05 (17.65)	4.02 (0.05-65.49)	35.31 (41.65)	19.52 (0.22-149.71)
Manual	R1	4.34 (4.45)	2.63 (0.38-15.44)	26.35 (26.62)	19.61 (1.92-90.47)
Manual	R2	4.82 (3.15)	4.60 (0.24-12.18)	27.75 (16.18)	28.70 (1.31-52.14)
T2 (N = 48)					
R1	R2	4.19 (4.28)	2.65 (0.27-20.39)	12.64 (13.45)	7.66 (1.03-67.41)
Model	Manual	11.60 (13.98)	5.40 (0.12-67.67)	29.06 (31.24)	14.36 (0.38-131.43)
Model	R1	11.57 (15.20)	6.40 (0.05-80.00)	29.10 (33.29)	18.06 (0.19-142.16)
Model	R2	10.92	5.07	26.61	15.15

		(14.38)	(0.04-73.35)	(31.41)	(0.20-138.36)
Manual	R1	6.47 (7.59)	3.81 (0.41-36.66)	18.74 (19.54)	10.56 (1.24-91.94)
Manual	R2	5.99 (6.53)	3.99 (0.16-33.19)	17.39 (16.36)	11.62 (0.35-63.71)
**T3 (N = 21)**					
R1	R2	14.36 (14.47)	7.82 (0.32-49.70)	20.02 (19.59)	12.18 (0.64-66.13)
Model	Manual	9.77 (12.03)	6.15 (0.18-48.34)	12.25 (15.01)	7.42 (0.24-60.30)
Model	R1	11.88 (12.77)	8.82 (1.59-55.00)	14.92 (15.73)	9.31 (1.79-71.59)
Model	R2	15.06 (13.57)	10.32 (1.26-48.60)	20.73 (18.98)	15.30 (1.90-72.95)
Manual	R1	11.38 (13.61)	7.62 (0.44-49.54)	14.70 (15.70)	12.08 (0.38-64.23)
Manual	R2	15.73 (15.90)	7.86 (0.26-48.79)	21.51 (21.58)	12.57 (0.47-80.80)

Supplemental Table 4 Legend: Absolute and relative error metrics are given for pairwise comparisons between the model segmentations, manual segmentations, Reviewer 1, and Reviewer 2 on the primary tumor longest dimension (LD) for T1, T2, and T3 tumors.

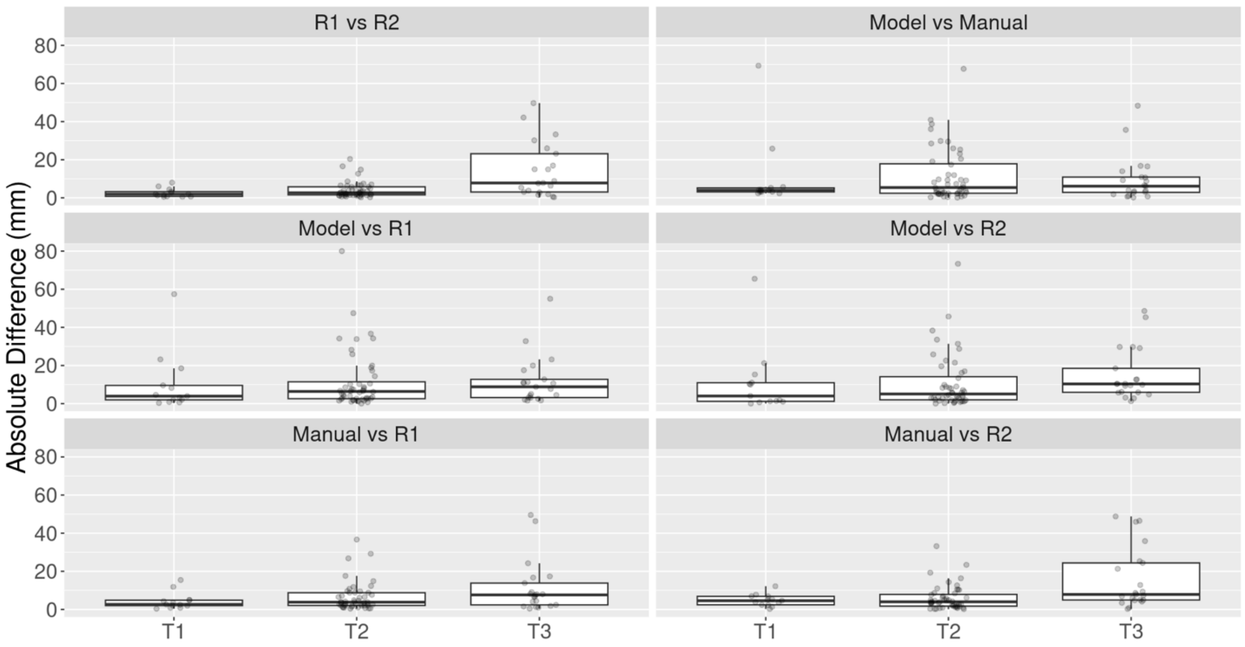

Supplemental Figure 3: Boxplots of Primary Tumor Longest Dimension Absolute Error by T stage. The absolute difference on primary tumor longest dimension between different measurement sources (Review 1 (R1), Reviewer 2 (R2), model segmentations, and manual segmentations) is shown stratified by T stage.

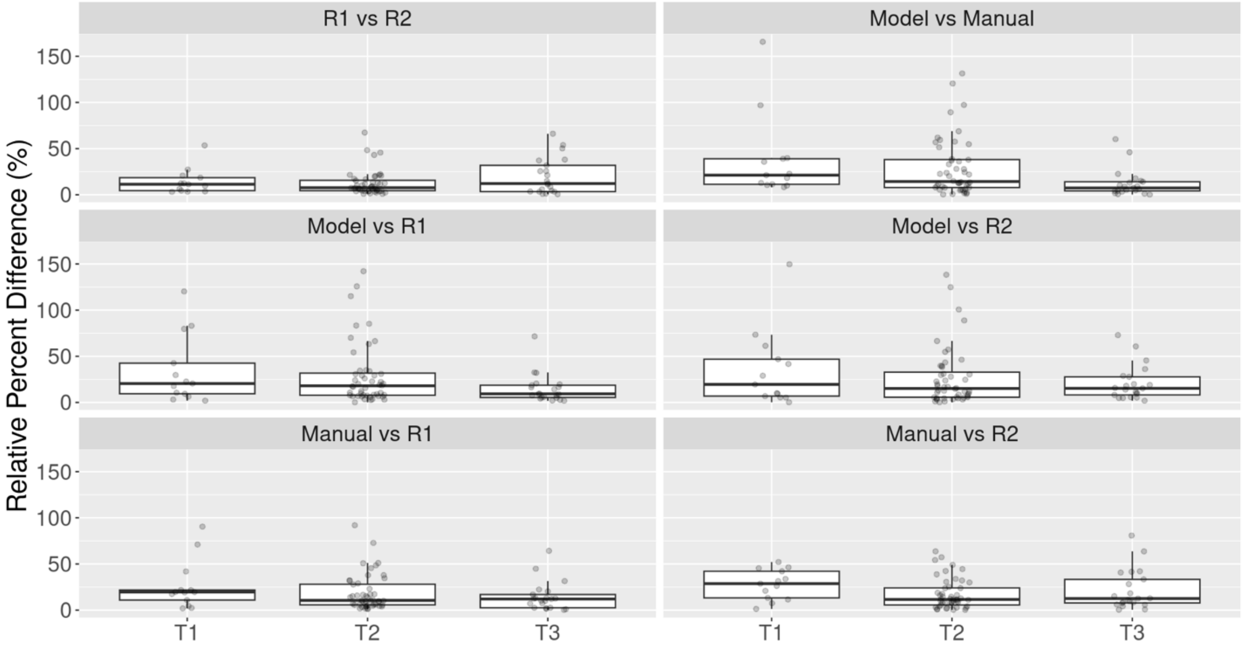

Supplemental Figure 4: Boxplots of Primary Tumor Longest Dimension Relative Percent Difference by T stage. The relative percent difference on primary tumor longest dimension between different measurement sources (Review 1 (R1), Reviewer 2 (R2), model segmentations, and manual segmentations) is shown stratified by T stage.

Performance by Cancer Subtype

We examined the longest dimension (LD) measurement error between the radiologists (R1 and R2), the manually verified AI-assisted annotations (Manual), and the TumorSight Viz segmentations (Model) stratified by cancer subtype. We grouped the cancer subtypes as invasive ductal carcinoma (IDC), invasive lobular carcinoma (ILC), and other. Of the 82 cases with primary tumor measurements from both radiologists as well as from the TumorSight Viz segmentation, 67 (82%) are IDC, 9 (11%) are ILC, and 6 (7%) are other cancer types. Absolute error and relative percent difference from pairwise comparisons of all measurement sources can be found in Supplemental Table 5 and are shown in Supplemental Figures 5 and 6.

Supplemental Table 5: Pairwise Measurement Error of Primary Tumor Maximum LD by Cancer Subtype

Primary Tumor Maximum LD					
Source		Absolute Error (mm)		Relative Percent Difference (%)	
Meas. 1	Meas. 2	Mean (SD)	Median (Range)	Mean (SD)	Median (Range)
IDC (N = 67)					
R1	R2	6.40 (9.41)	3.24 (0.27-49.70)	14.21 (15.23)	8.70 (0.99-67.41)
Model	Manual	10.04 (13.14)	4.24 (0.12-67.67)	23.14 (25.90)	12.85 (0.38-131.43)
Model	R1	11.10 (14.55)	5.95 (0.05-80.00)	23.89 (26.33)	16.36 (0.19-125.74)
Model	R2	11.77 (14.31)	6.17 (0.05-73.35)	24.29 (25.01)	14.80 (0.22-124.82)
Manual	R1	6.18 (8.19)	3.83 (0.38-49.54)	16.09 (17.27)	10.40 (0.38-91.94)
Manual	R2	7.55 (10.04)	4.67 (0.16-48.79)	18.38 (17.68)	11.80 (0.35-80.80)
ILC (N = 9)					
R1	R2	4.84 (5.76)	2.45 (0.65-14.95)	13.80 (13.83)	7.85 (3.29-45.67)
Model	Manual	8.01 (8.21)	3.13 (1.80-25.83)	23.78 (28.63)	12.86 (4.35-97.01)
Model	R1	7.98 (8.03)	4.50 (0.38-23.21)	26.07 (32.34)	10.04 (1.93-83.06)

Model	R2	6.65 (7.46)	4.64 (0.04-21.24)	20.65 (27.22)	5.75 (0.20-73.42)
Manual	R1	7.51 (7.86)	3.53 (0.49-24.22)	21.80 (21.17)	15.07 (1.24-71.06)
Manual	R2	7.98 (6.87)	7.32 (0.34-21.33)	23.30 (17.23)	25.06 (1.05-52.14)
**Other (N = 6)**					
R1	R2	10.61 (11.17)	6.92 (0.32-30.15)	23.02 (20.05)	19.19 (0.64-53.46)
Model	Manual	25.61 (24.39)	25.61 (0.18-69.31)	63.45 (65.22)	39.58 (0.24-165.70)
Model	R1	20.71 (20.14)	14.09 (1.59-57.46)	53.88 (60.70)	20.08 (2.12-142.16)
Model	R2	22.61 (22.38)	15.56 (2.93-65.49)	59.77 (65.74)	23.37 (5.85-149-71)
Manual	R1	20.76 (14.48)	14.60 (9.06-46.27)	46.11 (28.81)	44.34 (12.97-90.47)
Manual	R2	17.03 (15.67)	10.59 (3.83-45.96)	34.35 (18.32)	34.69 (12.81-63.66)

Supplemental Table 5 Legend: Absolute and relative error metrics are given for pairwise comparisons between the model segmentations, manual segmentations, Reviewer 1, and Reviewer 2 on the primary tumor longest dimension (LD) for IDC, ILC, and Other cancers.

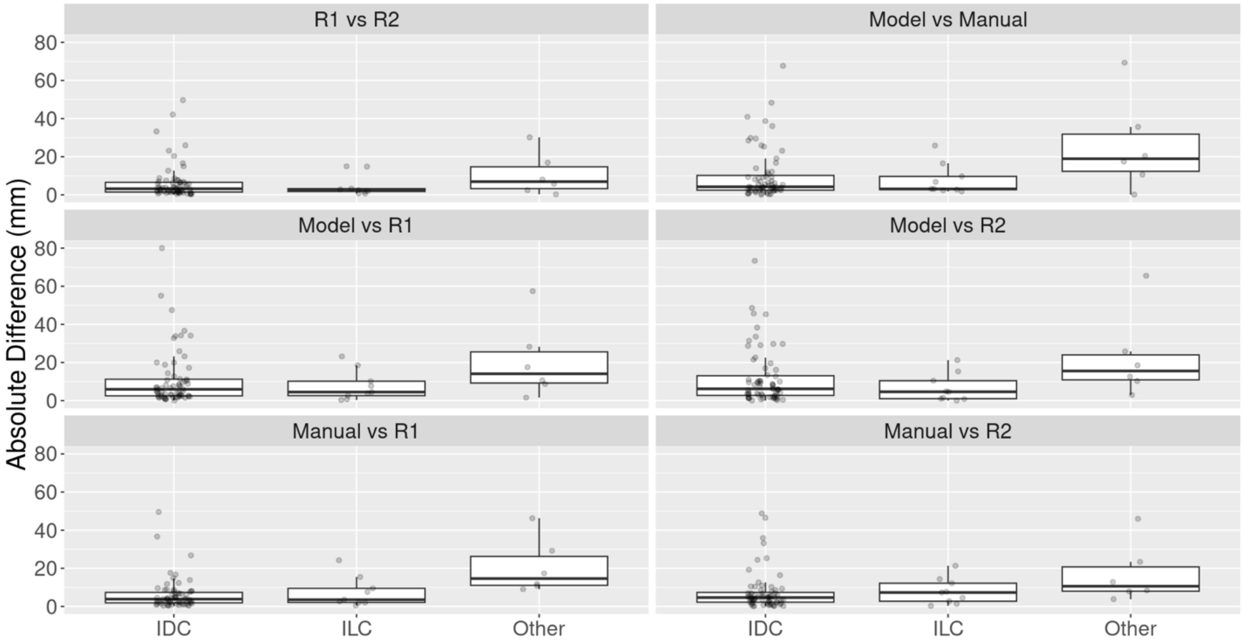

Supplemental Figure 5: Boxplots of Primary Tumor Longest Dimension Absolute Error by Cancer Type. The absolute difference on primary tumor longest dimension between different measurement sources (Review 1 (R1), Reviewer 2 (R2), model segmentations, and manual segmentations) is shown stratified by cancer type.

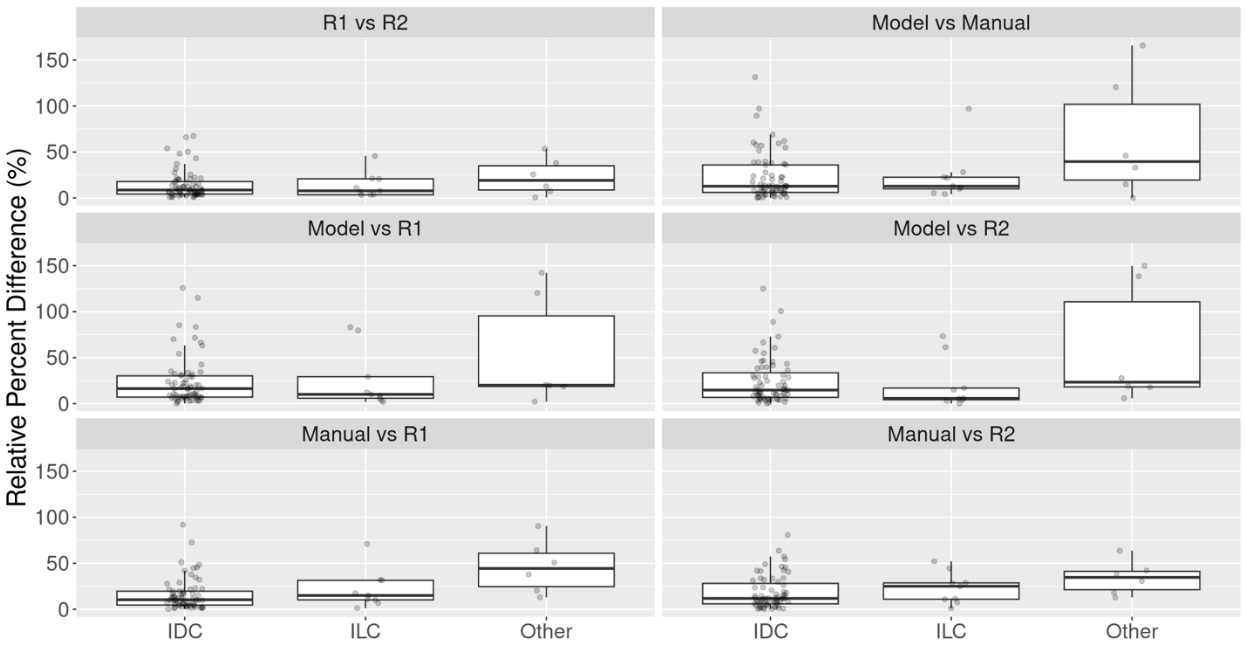

Supplemental Figure 6: Boxplots of Primary Tumor Longest Dimension Relative Percent Difference by T stage. The relative percent difference on primary tumor longest dimension between different measurement sources (Review 1 (R1), Reviewer 2 (R2), model segmentations, and manual segmentations) is shown stratified by cancer type.
